# Comparative Transcriptome Analysis Provides Novel Molecular Events for the Differentiation and Maturation of Hepatocytes during the Liver Development of Zebrafish

**DOI:** 10.3390/biomedicines10092264

**Published:** 2022-09-13

**Authors:** Yasong Zhao, Xiaohui Li, Guili Song, Qing Li, Huawei Yan, Zongbin Cui

**Affiliations:** 1 State Key Laboratory of Freshwater Ecology and Biotechnology, Institute of Hydrobiology, Chinese Academy of Sciences, Wuhan 430072, China; 2 Guangdong Provincial Key Laboratory of Microbial Culture Collection and Application, State Key Laboratory of Applied Microbiology Southern China, Institute of Microbiology, Guangdong Academy of Sciences, Guangzhou 510070, China; 3 College of Advanced Agricultural Sciences, University of Chinese Academy of Sciences, Beijing 100049, China; 4 Yangtze River Fisheries Research Institute, Chinese Academy of Fishery Sciences, Wuhan 430223, China

**Keywords:** zebrafish, liver, hepatocyte, transcriptome, signaling pathways

## Abstract

The liver plays an essential role in multiple biological functions including metabolism, detoxification, digestion, coagulation, and homeostasis in vertebrates. The specification and differentiation of embryonic hepatoblasts, the proliferation of hepatocytes, and the hepatic tissue architecture are well documented, but molecular events governing the maturation of hepatocytes during liver development remain largely unclear. In this study, we performed a comparative transcriptome analysis of hepatocytes that were sorted by flow cytometry from developing zebrafish embryos at 60, 72, and 96 hpf. We identified 667 up-regulated and 3640 down-regulated genes in hepatocytes between 60 and 72 hpf, 606 up-regulated and 3924 down-regulated genes between 60 and 96 hpf, and 1693 up-regulated genes and 1508 down-regulated genes between 72 and 96 hpf. GO enrichment analysis revealed that key biological processes, cellular components, and molecular functions in hepatocytes between 60 to 72 hpf, such as cell cycle, DNA replication, DNA repair, RNA processing, and transcription regulation, are mainly associated with the proliferation of hepatocytes. In addition to biological processes, cellular components, and molecular functions for cell proliferation, molecular functions for carbohydrate metabolism were enriched in hepatocytes during 72 to 96 hpf. KEGG enrichment analysis identified key signaling pathways, such as cell cycle, RNA degradation, ubiquitin-mediated proteolysis, ErbB and Hedgehog signaling, basal transcription factors, Wnt signaling, and glycan degradation, which are closely associated with cell proliferation or carbohydrate metabolism in hepatocytes between 60 to 72 hpf. Newly enriched signaling pathways in hepatocytes during 72 to 96 hpf include metabolisms of pyrimidine, purine, nicotinate and nicotinamide, *caffeine*, glycine, serine and threonine, ABC transporters, and p53 signaling that function in metabolisms of lipid, protein and energy, cellular secretion, or detoxification, indicating the functional maturation of hepatocytes between 72 to 96 hpf. These findings provide novel clues for further understanding the functional differentiation and maturation of hepatocytes during liver development.

## 1. Introduction

The liver is a vital organ with multiple biological functions, including metabolism, detoxification, digestion, and homeostasis in vertebrates [1]. As the metabolic center of the body, liver consists of multiple cell types in which hepatocytes (HCs) account for approximately 70% of the total liver cell population and carry out most functions of the liver, including the metabolism of lipids and drugs; storage of amino acids, iron, and glycogen; and production of clotting factors [2,3]. Therefore, the development, functions, and diseases of liver in vertebrates have been popular research topics [4,5,6].

Previous studies have revealed that molecular mechanisms of early liver development are conserved in vertebrates [7,8,9]. The factors encoding by homologous genes can regulate hepatic patterning in both zebrafish and mice [10,11]. Many highly-conserved and tightly-controlled signaling pathways, such as Wnt/β-catenin, Fgf, and Bmp signaling pathways play major roles during liver development [12,13,14]. A series of liver-specific factors, pan-endodermal factors, and factors from the surrounding mesodermal tissues together formed a genetic network and precisely control the process of liver organogenesis [15,16].

In mice, the differentiation of hepatoblasts initiates at approximately E11.5–E13.5 and terminates at E18.5 [15,17]. During this stage, Notch and TGFβ signals promote the maturation of cholangiocytes and inhibit the specification of hepatoblasts toward hepatocytes [18]. The maturation of hepatocytes is a gradual process that begins immediately after the differentiation of bipotential hepatoblasts and uninterruptedly until postnatally to develop the organizational architectural features of the liver [19]. Wnt/β-catenin signaling was responsible for the differentiation and expansion of hepatocytes, as well as managing the accomplishment of hepatocyte zonation. *Wnt1* and *Lhx2* deletion embryos showed an ectopic activation state with ECM deposition, fibrosis, and the smaller liver phenotype due to hepatocyte proliferation abnormalities [20]. Other factors that promote hepatocyte maturation include the cytokine oncostatin M (OSM), hepatocyte growth factor (HGF), and the continuing inhibition of Notch and TGF signaling [21,22]. 

Zebrafish (*Danio rerio*) is an excellent model organism to study both liver development and regeneration [23,24]. The zebrafish liver, as other vertebrates, develops from the endoderm, but it lacks hematopoietic function, and mutations in genes associated with early liver development do not result in hematopoietic dysfunction [25]. 

The process of zebrafish liver organogenesis can be divided into four main phases including hepatoblasts specification, budding, differentiation, and outgrowth [26,27]. Zebrafish liver morphogenesis started at 22 hpf and ended at 96 hpf, which can be distinguished by morphological changes and the expression patterns of molecular markers, such as *hhex*, *proxl*, *foxa3*, and *gata6* [28]. The mesodermal signals, such as Fgf, Bmp, and Wnt signaling are closely involved in the control of hepatoblast specification, which can be marked by *prox 1* and *hhex* at 22 hpf in zebrafish [29,30]. The differentiation and maturation of hepatocytes and outgrowth of zebrafish liver occur between 50 and 96 hpf, which are marked by *vitamin D binding protein* (*gc*), *fatty acid binding protein 10a* (*fabp10a*), *ceruloplasmin* (*cp*), and the dramatic increase in liver size to establish the liver functions [31,32,33]. Moreover, Hnf members are a group of transcription factors that are enriched in the liver when compared to other organs, of which Hnf1 and Hnf6 are essential factors in hepatocyte maturation and outgrowth [34,35]. Although a large body of studies have focused on liver development, including the specification of hepatoblasts, the proliferation of hepatocytes, and the hepatic tissue architecture, the molecular events underlying the differentiation of hepatocyte functions during outgrowth and maturation of liver remain largely unclear.

Generation of multiple transgenic zebrafish lines carrying a fluorescent liver, such as *Tg(fabp10a:dsRed;ela3l:EGFP)* zebrafish [36], which can express the red fluorescent protein under hepatocyte-specific promoter *fabp10a* (fatty acid binding protein 10a, also called *l-fabp*), allows the *dynamic* study of early developmental of liver in vivo [37]. In this study, hepatocytes with red fluorescence were sorted with a flow cytometry from developing embryos at 60, 72, and 96 hpf of the *Tg(fabp10a:dsRed;ela3l:EGFP)* line, followed by a high-throughput RNA-seq analysis. The comparison of transcriptome profiling for hepatocyte populations from three time points revealed multiple signaling pathways and biological processes that are associated with the functional differentiation and maturation of hepatocytes.

## 2. Materials and Methods

### 2.1. Maintenance of Zebrafish

Wild type AB strain and *Tg(fabp10a:dsRed;ela3l:EGFP)* line were maintained and bred in a circulating water system at 28 °C. Embryos at different developmental stages were determined according to hours post-fertilization (hpf). The collection and culture of embryos were performed following the previous methods [38].

### 2.2. Preparation of Zebrafish Liver Cell Suspension

Approximately 4000 embryos were collected from the hybrid offspring of wild-type (WT) females and transgenic zebrafish males with a red fluorescent liver. The zebrafish larvae at 20 hpf were cultured in 0.3 × Danieau’s solution (17 mM NaCl, 0.2 mM KCl, 0.12 mM MgSO_4_, 0.18 mM Ca(NO_3_)_2_, 1.5 mM HEPES, pH 7.6) containing 0.003% (w:v) 1-phenyl 2-thiourea (PTU). At 60, 72 or 96 hpf, after anesthetized in 0.3 × Danieau’s solution containing 0.016% Tricaine mesylate (Tricaine methanesulfonate, MS-222) (Sigma, St. Louis, MO, USA), approximately 2000 fluorescent zebrafish embryos were selected under a fluorescence microscope and washed three times with 0.3 × Danieau’s solution. Then, 50 larvae fish were put into a 2 mL centrifuge tube, followed by the removal of excess liquid, addition of 1 mL trypsin-EDTA solution, digestion in a water bath at 28 °C for 10 min, and pipetting up and down with a P200 pipette every 3 min. After digestion, Hi-FBS at a final concentration of 5% was added to stop the reaction and the tubes were placed in a water bath at 28 °C for 5–7 min. Next, the above digestion solution was filtered into a new 1.5 mL centrifuge tube using a 200-mesh sieve and then centrifuged at 310✕ *g* for 5 min. Finally, the zebrafish hepatocytes were resuspended in PBS (Hyclone, #SH30256.01, Logan, UT, USA). All steps of the experiments were performed on ice unless otherwise noted [39].

### 2.3. Isolation of Fluorescent Cells in Zebrafish Liver

Hoechest 33342 (Beyotime, #C1025, Shanghai, China) is a membrane-permeable fluorescent dye, which shows a bright-blue signal in the nucleus of apoptotic cells. The wavelength of the fluorescent dye is different from the wavelength of the red fluorescence of hepatocytes, which is convenient for removing dead cells before sorting as previously described [40]. After primary sorting, recovery, and secondary sorting with a flow cytometry (BD Biosciences, Franklin Lakes, NJ, USA), the cells were sorted into FACSmax solution (AMSBIO, Abingdon, UK) and 10–20 µL was used for cell counting with a Neubauer cell counter slide to check the purity of desired cells. Finally, we isolated red-fluorescent hepatocytes with a purity of more than 95%. Samples for RNA-seq analysis at each time point have three replicates.

### 2.4. RNA Extraction

RNA extraction was conducted immediately after the fluorescence-activated cell sorting (FACS). Sorted hepatocytes from three time points were transferred directly into the lysis buffer supplied by the RNeasy Micro Kit (Qiagen, #74004, Düsseldorf, Germany) and then performed according to the manufacturer’s instructions [41]. RNA was isolated from cell lysates right away. RNA degradation and contamination were detected using 1% agarose gel electrophoresis. A NanoPhotometer NP60 Spectrometer (Implen GmbH, München, Germany) was used to measure the purity and concentration of RNA. Following the manufacturer’s instructions, the Direct-zol RNA MiniPrep Kit (ZYMO RESEARCH, #R2050, Irvine, CA, USA) was used to purify RNA [42].

### 2.5. Library Construction and High-Throughput Sequencing

The construction of libraries and high-throughput sequencing were performed using an Illumina Genome Analyzer IIx platform (GA IIx, Illumina, San Diego, CA, USA) in the Analysis and Testing Center of the Institute of Hydrobiology, Chinese Academy of Sciences (http://www.ihb.ac.cn/fxcszx/, accessed on 8 November 2021). Purified mRNA samples were fragmented into small pieces and double-stranded cDNA was synthesized using random hexamer primers. Synthetic cDNAs were end-repaired, phosphorylated, 3’-adenylated, adaptor-ligated, and PCR-amplified to construct sequencing libraries. Three independent biological replicates for samples from three time points were used for library construction. 

### 2.6. Bioinformatic Analysis

The raw reads contain adapters or low-quality bases. Therefore, the reads were filtered with PRINSEQ (version 0.20.4, Schmieder R, San Diego, CA, USA) to obtain high quality clean reads [43]. Based on the reference-based approach, we used StringTie (version 1.3.1, StringTie, Baltimore, MD, USA) software to assemble the mapped reads of each sample [44,45]. The differentially expression analysis of data for samples between two time points was performed following the previous methods [46]. Genes with a fold change ≥ 2 and a q-value ≤0.05 were considered significantly and differentially expressed. After comparison with the zebrafish reference genomic data (*Danio rerio*. GRCz11, version-103), an enrichment analysis of differentially expressed genes (DEGs) identified the signal transduction pathways and metabolic pathways. GO (Gene ontology) enrichment analyses were conducted using Cytoscape-v3.8.2 plugins ClueGO-v2.5.8. KEGG (Kyoto Encyclopedia of Genes and Genomes) enrichment analyses were performed using the clusterProfiler package. Calculation and creation of Jaccard Coefficient (JC) and network of hub genes and pathways were performed as previously described [47,48].

### 2.7. Statistical Analysis

Statistical analysis of data was performed using GraphPad Prism 8.3.0 software (GraphPad Software, San Diego, CA, USA).

## 3. Results

### 3.1. The Isolation of Zebrafish Hepatocytes

**Figure 1:**
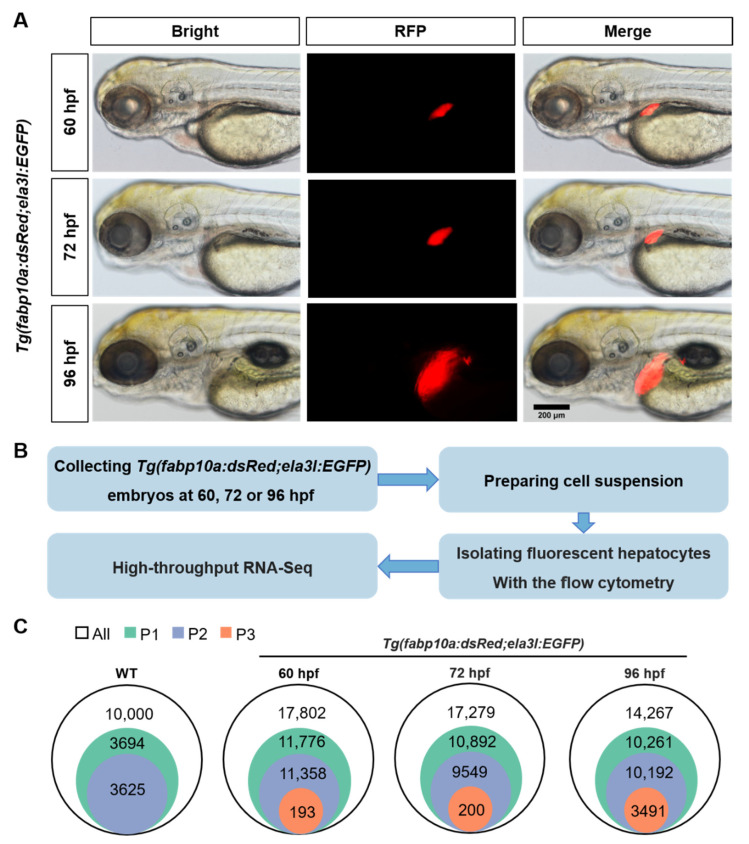
The isolation of zebrafish hepatocytes. (**A**) The size changes of red-fluorescent liver at 60, 72, and 96 hpf in transgenic zebrafish *Tg(fabp10a:dsRed;ela3l:EGFP)*. Scale bar, 200 μm. (**B**) The technical roadmap for isolation of zebrafish hepatocytes. (**C**)The cell populations of hepatocytes sorted from *Tg(fabp10a:dsRed;ela3l:EGFP)* embryos at 60, 72 and 96 hpf.

The transgenic zebrafish line *Tg(fabp10a:dsRed;ela3l:EGFP)* was used to isolate hepatocytes labeled by DsRed, a red-fluorescent protein. During embryonic development, the size of developing zebrafish liver increased rapidly from 60 to 96 hpf as shown by the red fluorescence area (Figure 1A). Developing embryos at 60, 72, or 96 hpf were digested into individual cells. The embryonic cells were sorted with flow cytometry to obtain dsRed-expressing hepatocytes at 60, 72, or 96 hpf, followed by RNA-seq (Figure 1B). Population 1 (P1) represents a population of cells that have been removed from cellular debris. After primary sorting and recovery, we obtained population 2 (P2), which excluded the dead cells stained with Hoechest. The number of sorted hepatocytes continued to increase with the outgrowth of liver from 60 to 96 hpf (Supplementary Figure S1). We collected cell population 3 (P3) at 60, 72, or 96 hpf, in which the red-fluorescent hepatocytes take up to more than 95%. The number of sorted hepatocytes for further analysis were 193, 200, and 3491, which continued to increase with the outgrowth of liver from 60 to 96 hpf (Figure 1C)

### 3.2. Quality Analysis of the Transcriptome Data for Zebrafish Hepatocytes

To understand biological processes, cellular functions and signaling pathways that are associated with the functional differentiation and maturation of hepatocytes in developing embryo at 60, 72, and 96 hpf, nine samples of P3 hepatocytes were sorted for construction of RNA libraries and subsequcent high-throughput RNA-seq sequencing and samples at each time point contain three independent biological replicates.

**Figure 2:**
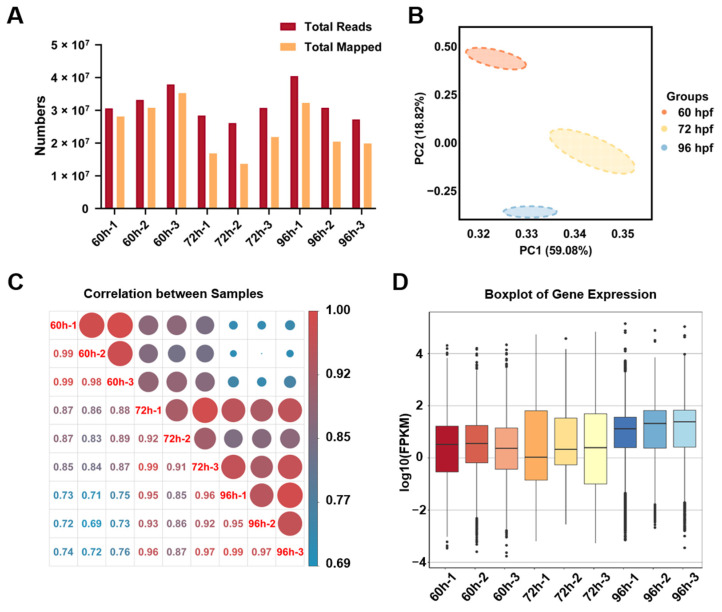
The quality analysis of transcriptome data for zebrafish hepatocytes. (**A**) The RNA-seq data of sorted liver cells at three time points. (**B**) The principal component analysis (PCA) of samples at three time points. (**C**) The Pearson’s correlation coefficient matrix of RNA-seq between samples at three time points. (**D**) The boxplots of gene expression in samples at three time points.

RNA-seq analysis generated 27.2249–40.4443 million pairs (M) of total reads for each of the samples and approximately 72.32–77.77% of the processed reads were mapped to the reference genome of zebrafish and unique mapped genes accounted for more than 90% of total mapped genes (Figure 2A). The Q20 and Q30 of the three groups at 60, 72, and 96 hpf were all above 85% and the GC content was 46.89% at 60 hpf, 49.31% at 72 hpf and 46.77% at 96 hpf, respectively (Table 1). These data demonstrated the relatively high quality of the RNA sequencing. The first (PC1) and second (PC2) principal component analysis (PCA) showed a variation of 59.08% and 18.82%, indicating a clear separation of genes at different time periods during the early development of the zebrafish liver (Figure 2B).

To evaluate the similarity between samples collected at the same time points, we calculated the correlation between different samples. The closer correlation coefficient between samples gets to 1, the higher similarity between samples is, and the fewer differentially expressed genes between samples. We found that Pearson’s correlation between samples at the same time point was 0.91–0.99 and the correlation between 60 and 72 hpf was higher than between 60 and 96 hpf (Figure 2C), which was consistent with the data of PCA. Boxplot comparison of the distributions of gene expression data after normalization showed that the means and ranges of gene expression in each sample exhibit a uniformity of the expression distribution (Figure 2D). 

### 3.3. Differentially Expressed Genes in Hepatocytes of Developing Liver

**Figure 3:**
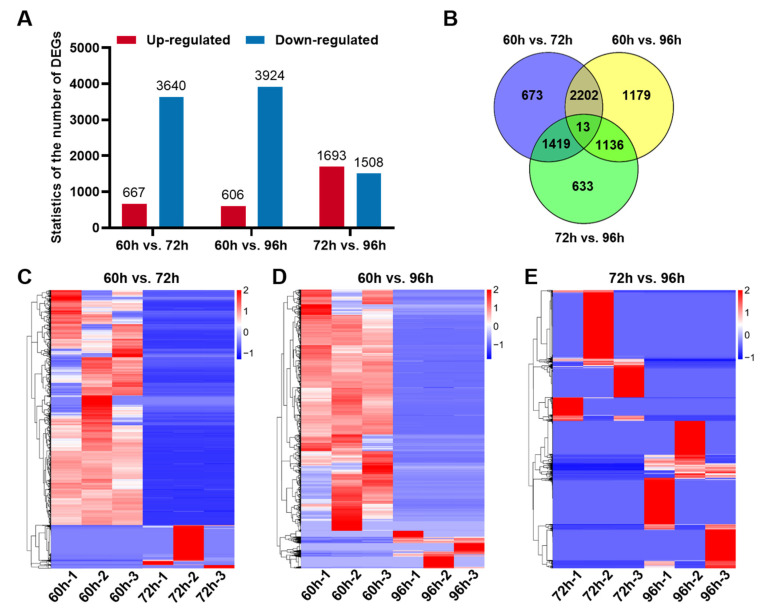
The differentially expressed genes analysis in early liver developmental stages. (**A**) Statistics of differentially expressed genes (DEGs) in hepatocytes between two time points. (**B**) The Venn diagram analysis of DEGs in hepatocytes among three time points. Heatmaps of DEGs between two time points, including 60 and 72 hpf (**C**), 60 and 96 hpf (**D**), as well as 72 and 96 hpf (**E**).

We then performed a Venn diagram analysis to identify differentially expressed genes (DEGs) for three time periods 60–72 hpf, 72–96 hpf, and 60–96 hpf in heptocytes of developing liver. A total of 7255 DEGs were found, including up-regulated (log2foldchange ≥ 1, adjusted *p*-value ≤ 0.05) and down-regulated (log2foldchange ≤ −1, adjusted *p*-value ≤ 0.05) between two time points were listed in Supplementary Table S1. We found 667 up-regulated and 3640 down-regulated genes in heptocytes betweent 60 and 72 hpf, 1693 up-regulated and 1508 down-regulated genes between 72 and 96 hpf, and 606 up-regulated and 3924 down-regulated genes between 60 and 96 hpf (Figure 3A). Among these DEGs, 673 DEGs were specifically detected between 60 and 72 hpf, 633 DEGs between 72 and 96 hpf, and 1179 DEGs between 60 and 96 hpf (Figure 3B). 

As shown in cluster heatmaps, a striking difference in the expression of genes can be found in hepatocytes between two time points and most DEGs at 72 hpf or 96 hpf were down-regulated in comparison with the expression of corresponding genes at 60 hpf (Figure 3C–E), suggesting that the differentiation of hepatocyte functions occurred from 60 to 96 hpf.

### 3.4. GO Enrichment Analysis of Specifically Expressed Genes in Hepatocytes during Liver Development

**Figure 4:**
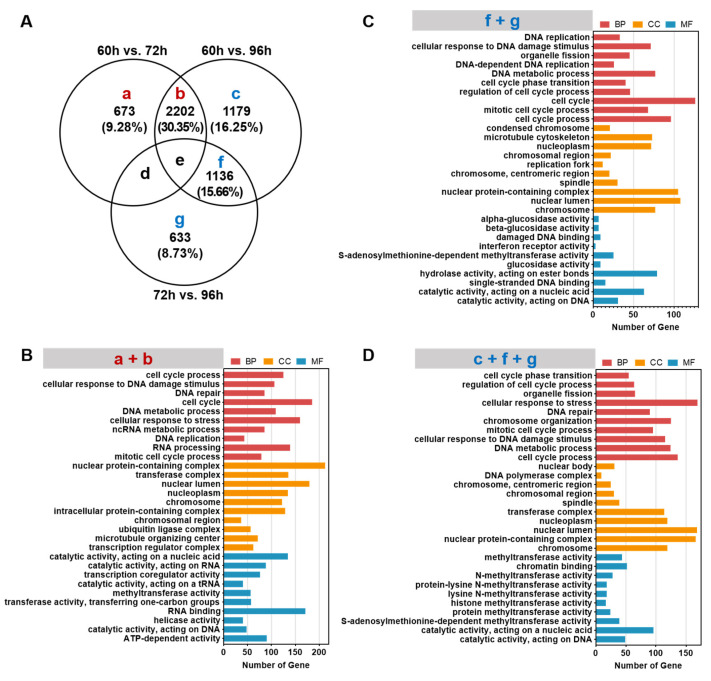
GO enrichment analysis of genes specifically expressed during different time periods of liver development. (**A**) The Venn diagram analysis of differentially expressed genes. a-g: different letters represent genes specifically expressed in different Venn groups. (**B**) GO enrichment analysis of genes specifically expressed in groups a and b that represent a class of genes specifically expressed in hepatocytes from 60 to 72 hpf. (**C**) GO enrichment analysis of genes specifically expressed in groups f and g that represent a class of genes specifically expressed in hepatocytes from 72 to 96 hpf. (**D**) GO enrichment analysis of genes specifically expressed in groups c, f, and g that represent all differentially expressed genes in hepatocytes between 60–72 hpf and 72–96 hpf.

To further explore the differences in biological processes (BP), cellular composition (CC) and molecular functions (MF) in heptocytes from 60 to 96 hpf, all DEGs were divided into seven groups (a–g) (Figure 4A; Table S2). DEGs in groups a and b represent genes specifically expressed in hepatocytes from 60 to 72 hpf, which account for 39.63% of all DEGs. DEGs in groups f and g were specifically expressed in heptocytes from 72 to 96 hpf, which account for 24.39% of total DEGs. DEGs in groups c, d, and e showed no significance in hepatocytes from 60 to 72 hpf, but DEGs in group c stand for a significant difference in hepatocytes from 60 to 96 hpf, suggesting that DEGs in group c are also associated with functional differention of hepatocytes. Thus, GO enrichment analysis of DEGs in groups (a + b, f + g, c + f + g, d + e, b + e, c + f) were performed. 

Top 10 GO terms and genes related to biological processes, cellular composition and molecular function in different groups were listed in Supplementary Table S3. The top 10 GO terms enriched from genes specifically expressed in groups a and b are primarily cell cycle, cellular response to stress, DNA replication, DNA repair and RNA processing in BP, nuclear protein-containing complex and nuclear lumen in CC, and catalytic activity on DNA and RNA and RNA binding in MF (Figure 4B), which are closely associated with cell proliferation. Most BPs, CCs, and MFs related to cell proliferation were also shared in hepatocytes among groups (d + e, b + e, c + f) at 60–72 hpf, 72–96 hpf and 60–96 hpf (Supplementary Figure S1). Moreover, lipid biosynthetic process, ATP dimethylallyltransferase activity and ADP dimethylallyltransferase activity were shared in hepatocytes between 60–72 hpf and 72–96 hpf (groups d + e, Supplementary Figure S2). These data suggest that functions of cell proliferation, lipid synthesis, and energy metabolism are developed in hepatocytes from 60 to 96 hpf.

In addition to DEGs related to cell proliferation, the genes specifically expressed in groups f and g were associated with molecular functions of hydrolase activity on ester bonds, glucosidase activity for carbohydrate metabolism and S-adenosylmethionine-dependent methyltransferase activity in hepatocytes between 72 to 96 hpf (Figure 4C). Moreover, multiple methyltransferase activities were enriched in hepatocytes from 72 to 96 hpf (groups f + g + c, Figure 4D) and from 60 to 96 hpf (groups c + f, Supplementary Figure S2). 

### 3.5. Enrichment of KEGG Pathways and Hub Genes Associated with the Proliferation and Maturation of Hepatocytes

**Figure 5:**
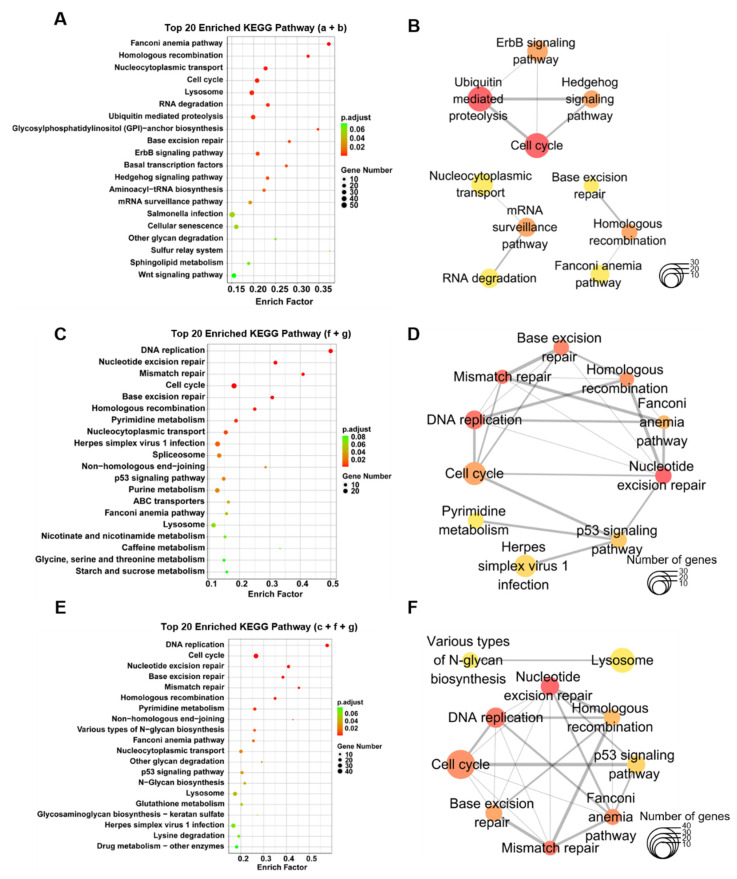
Hub signaling pathways from KEGG enrichment analysis of genes specifically expressed in hepatocytes at different stages. Dot plots of KEGG signaling pathways (**A**) and networks of top 10 hub pathways (**B**) for genes specifically expressed from 60 to 72 hpf (a and b). Dot plots of KEGG signaling pathways (**C**) and networks of top 10 hub pathways (**D**) for genes specifically expressed from 72 to 96 hpf (f and g). Dot plots of KEGG signaling pathways (**E**) and networks of top 10 hub pathways (**F**) for genes specifically expressed from 60 to 96 hpf (c, f and g). Node color stands for the enrichment p-value in the pathway.

KEGG enrichment analysis was performed to reveal the functional characteristics of DEGs in hepatocytes of developing liver (Supplementary Figure S3; Supplementary Table S4). The distances between different signaling pathways were calculated by Jaccard coefficient according to the proportion of shared genes to obtain the signaling networks of DEGs in different groups. Most of genes specifically expressed in groups a and b from 60 hpf to 72 hpf were enriched in signaling pathways, such as cell cycle, RNA degradation, ubiquitin-mediated proteolysis, ErbB, Hedgehog, basal transcription factors, Wnt, and glycan degradation (groups a + b; Figure 5A), which are closely associated with cell proliferation or carbohydrate metabolism in hepatocytes between 60 to 72 hpf. The ErbB signaling pathway, ubiquitin mediated proteolysis, and cell cycle were the top hub pathways in the network of KEGG enrichment signaling pathways for groups a and b (Figure 5B; Table 2; Supplementary Table S5).

In addition to signaling pathways for cell proliferation, newly enriched signaling pathways in hepatocytes between 72 to 96 hpf include metabolisms of pyrimidine, purine, nicotinate and nicotinamide, caffeine, glycine, serine and threonine, ABC transporters, and p53 signaling (groups f + g; Figure 5C), which function in metabolisms of lipid, protein and energy, cellular secretion, and detoxification, indicating the functional maturation of hepatocytes between 72 to 96 hpf. The top hub pathways include the cell cycle, DNA replication, pyrimidine metabolism, and p53 signaling (Figure 5D; Table 3; Supplementary Table S5). Similar signaling pathways were overrepresented in genes specifically expressed in groups c, f, and g (Figure 5E) and the top hub signaling pathways include various types of N-glycan biosynthesis, DNA replication and repair, and p53 signaling (Figure 5F; Table 4; Supplementary Table S5).

**Figure 6:**
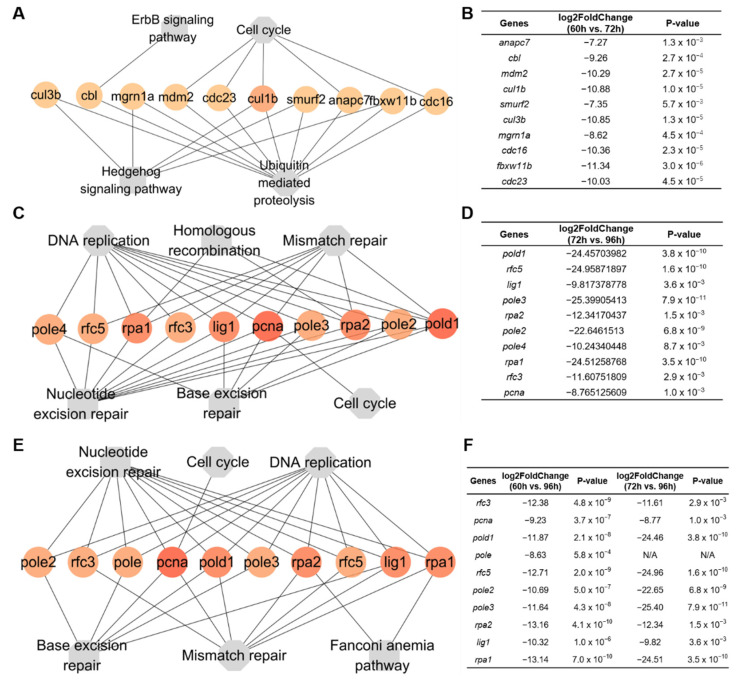
Hub genes within KEGG pathways. (**A**) Networks of 10 hub genes mapped to top four pathways in groups a and b from 60 to 72 hpf. (**B**) The fold changes of hub genes in groups a and b. (**C**) Networks of 10 hub genes mapped to top 6 pathways from 72 to 96 hpf in groups f and g. (**D**) The fold changes of hub genes in groups f and g. (**E**) Networks of 10 hub genes mapped to top 6 pathways from 60 to 96 hpf in groups c, f, and g. (**F**) The fold changes of hub genes in groups c, f, and g.

The hub genes of these KEGG pathways were examined with CytoHubba. In hepatocytes from 60 to 72 hpf, the hub genes (cul3b, cbl, mgrn1a, mdm2, cdc23, cul1b, smurf2, anapc7fb, xw11b, and cdc16) in groups a and b were clustered into ErbB signaling pathway, cell cycle, hedgehog signaling pathway, and ubiquitin-mediated proteolysis (Figure 6A,B). In hepatocytes from 72 to 96 hpf, the hub genes (pole4, rfc5, rpa1, rfc3, lig1, pcna, pole3, rpa2, pole2, and pold1) in groups f and g were clustered into DNA replication, homologous recombination, mismatch repair, nucleotide excision repair, base excision repair and cell cycle (Figure 6C,D). Similar to those in groups f and g, the hub genes (pole2, rfc3, pole, pcna, pold1, pole3, rpa2, rfc5, lig1, and rpa1) in groups c, f, and g were clustered into nucleotide excision repair, cell cycle, DNA replication, base excision repair, mismatch repair, and fanconi anemia pathway (Figure 6E,F). 

### 3.6. Dynamic Changes of DEGs in Hepatocytes during Liver Development

**Figure 7:**
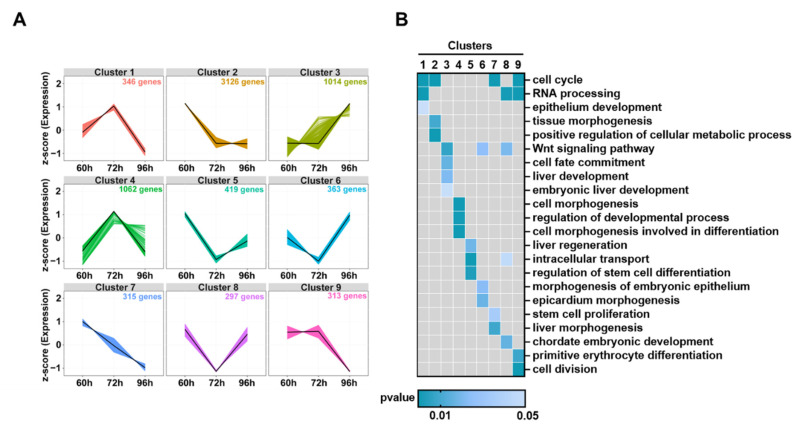
Dynamic changes of DEGs in hepatocytes from 60 to 96 hpf (**A**) Hierarchical clustering of changes in DEGs. (**B**) Functional annotations of different clusters by GO analysis. Heatmaps of biological processes were displayed according to their statistical significance (*p* < 0.05) and locations in the GO tree.

The transcriptome data were normalized by z-score and analyzed with fuzzy c-means clustering to classify the dynamic trends of DEGs in hepatocytes at 60, 72, and 96 hpf during liver development of zebrafish. The 7255 DEGs from 3 time periods 60–72 hpf, 72–96 hpf, and 60–96 hpf in heptocytes were categorized into 9 distinct clusters of which each cluster exhibited distinct expression patterns (Figure 7A).

GO enrichment analysis revealed that genes of nine clusters were associated with distinct biological processes (Figure 7B; Supplementary Table S6). DEGs in clusters 1 (*n* = 346, 4.8%) and 4 (*n* = 1062, 19.4%) were upregulated at 72 hpf and then gradually downregulated at 96 hpf in comparison with those at 60 hpf. DEGs in cluster 1 were enriched in biological processes of cell cycle, RNA processing, and epithelium development, while DEGs in cluster 4 were overrepresented in biological processes of cell morphogenesis, regulation of developmental process, and cell morphogenesis involved in differentiation.

DEGs in clusters 2 (*n* = 3126, 43.1%) were significantly downregulated at 72 hpf and maintained at a low expression level at 96 hpf when compared to those at 60 hpf. These DEGs are involed in cell cycle, tissue morphogenesis, and positive regulation of cellular metabolic process.

DEGs in cluster 3 (*n* = 1014, 14%) were upregulated at 96 hpf when compared with those at 60 and 72 hpf, which were associated with Wnt signaling pathway, cell fate commitment and liver development. DEGs in Clusters 5 (*n* = 419, 5.8%), 6 (*n* = 363, 5.0%) and 8 (*n* = 297, 4.1%) showed a “V” pattern of expression from 60 to 90 hpf, which were specifically associated with liver regeneration, intracellular transport, regulation of stem cell differentiation, Wnt signaling pathway, epicardium morphogenesis, and RNA processing. DEGs in Cluster 7 (*n* = 315, 4.3%) were continually downregulated from 60 to 96 hpf, which were highly enriched in cell cycle, stem cell proliferation and liver morphogenesis. DEGs in cluster 9 (*n* = 313, 4.3%) were almost unaltered from 60 to 72 hpf but downregulated from 72 to 96 hpf and these DEGs were associated with cell cycle, RNA processing, cell division, and primitive erythrocyte differentiation.

The process of cell cycle process appeared in four clusters (1, 2, 7 and 9), RNA processing in three clusters (1, 8, and 9), Wnt signaling pathway in three clusters (3, 6, and 8), and intracellular transport in two clusters (5 and 8), indicating that these biological processes are important in the proliferation and functional maturation of hepatocytes from 60 to 96 hpf.

## 4. Discussion

The liver is an essential organ in the body and performs a number of crucial activities, such as detoxification, metabolism, and homeostasis in vertebrates [1]. Liver diseases are becoming a worldwide problem that is threatening the health of humans [49]. Zebrafish (*Danio rerio*) are now commonly used in research on embryonic development, liver regeneration, and diseases [50,51]. In zebrafish, liver is an accessory organ of the foregut and liver morphogenesis can be divided into four phases, including the specification of hepatoblasts, the budding, differentiation, and outgrowth of hepatocytes [52,53]. The budding phase occurs at 24 hpf and ends at 50 hpf to form the hepatic duct. During the subsequent growth phase, the size, shape, and placement of liver began to extend across the midline ventral to esophagus and forms the architecture [54]. Studies in mammals indicated that liver development began with the appearance of liver buds, to the formation of liver progenitor cells, followed by the proliferation, differentiation, and migration of hepatic progenitor cells, and finally to the formation of liver, undergoing a complex process of cell signal regulation [12,21,53]. Furthermore, extrinsic signaling pathways and cell-autonomous transcription factors tightly regulate liver organogenesis [55]. Although the developmental patterns of liver in vertebrates are well established, biological processes and signaling pathways controlling the proliferation and maturation of hepatocytes remain largely unknown. In this study, we isolated hepatocytes from the development of embryos of *Tg(fabp10a:dsRed;ela3l:EGFP)* zebrafish at 60, 72, and 96 hpf and performed a comparative transcriptome analysis of these three hepatocyte populations. We identified a large number of DEGs, which are overrepresented in processes and signaling pathways associated with hepatocyte proliferation and function maturation. 

A previous study with inflammation models CCl4 and partial hepatectomy has shown that HNF4, CAR, and Krüppel-like factors MafF and ELK1 were conserved as key regulators of hepatoblasts [56]. From 60 to 90 hpf, many GO terms were associated with the proliferation of hepatocytes, such as cell cycle, cellular response to stress, DNA replication, DNA repair and RNA processing in BP, nuclear protein-containing complex and nuclear lumen in CC, and catalytic activity on DNA and RNA binding in MF. In addition to cell proliferation, lipid biosynthetic process, ATP dimethylallyltransferase activity, and ADP dimethylallyltransferase activity were shared in hepatocytes between 60–72 hpf and 72–96 hpf, indicating that hepatocytes from 60 to 90 hpf are still proliferating and functions of lipid synthesis and energy metabolism are established in hepatocytes from 60 to 96 hpf.

The liver development process involved in many pathways, such as bone morphogenetic protein (BMP), transforming growth factor β (TGFβ), Wnt, and Hippo and Notch signaling pathways in mammals [21]. The Wnt signaling pathway tightly controls embryogenesis, including hepatobiliary development, maturation, and zonation, and it can increase glucose metabolism in hepatocellular carcinoma cells [12,57]. The Wnt signal inhibitor IWR-1 can also significantly influence the development of zebrafish liver, which leads to liver dysplasia [58]. In this study, KEGG enrichment analysis indicated that most genes specifically expressed in hepatocytes from 60 to 72 hpf were enriched in signaling pathways, such as cell cycle, RNA degradation, ubiquitin-mediated proteolysis, ErbB, Hedgehog, basal transcription factors, Wnt, and glycan degradation. The ErbB family of proteins consist of four protein kinases involved in multiple signaling pathways, such as cell proliferation, differentiation, and apoptosis. Overexpression of ErbB2 promotes breast cancer cells to grow rapidly [59]. Moreover, a previous study has revealed that smn1, gemin3, and gemin5 were linked to a common set of genetic pathways, such as ErbB and tp53 pathways, which can affect the regeneration of liver [60]. Therefore, the liver between 60 and 72 hpf continues to grow and hepatocytes have developed the function of carbohydrate metabolism.

Metabolisms of lipid, protein, and energy were found to be closely related to the establishment of hepatocyte functions, which can prevent the accumulation of lipid droplets and provide the nutrients required in this process [16,61,62]. Albumin and urea secretion, glycogen storage, and metabolic activity of cytochrome P450 enzymes represent functional features of mature hepatocytes [18,63]. Genome-wide characterization of ESC-derived hepatocyte-like cells indicated that some genes are associated with metabolic processes such as small molecule metabolic processes or secondary metabolic processes [64]. Some transcription factors, such as FOXA1/2/3, HNF4α, and CEBPA can maintain hepatocyte maturation through a combined action [65]. In this study, we found that, in addition to signaling pathways for cell proliferation and DNA replication, newly enriched signaling pathways in hepatocytes between 72 to 96 hpf include metabolisms of pyrimidine, purine, nicotinate and nicotinamide, caffeine, glycine, serine and threonine, ABC transporters, and p53 signaling, which are known to function in metabolisms of lipid, protein and energy, cellular secretion, and detoxification. Moreover, the genes specifically expressed in hepatocytes from 72 to 96 hpf were enriched in molecular functions of hydrolase activity on ester bonds, glucosidase activity for carbohydrate metabolism, and *S-adenosylmethionine-dependent* *methyltransferase activity*. Thus, hepatocytes between 72 to 96 hpf are functionally matured.

To further understand the regulatory mechanisms of hepatocyte maturation, we classified DEGs into nine dynamic clusters by z-score standardization and fuzzy c-means clustering analysis. We found that several important pathways for embryonic development function during hepatocyte maturation from 60 to 96 hpf. For example, the Wnt signaling pathway is known to function in liver development [12] and DEGs, such as *wnt7bb*, *rspo3*, *wnt6b*, *tmem88a*, and *wnt2ba* are enriched in Cluster 3 (*n* = 1014, 14%), 6 (*n* = 363, 5.0%) and 8 (*n* = 297, 4.1%) in hepatocytes from 60 to 90 hpf. Meanwhile, tissue morphogenesis, liver morphogenesis, and liver regeneration were found in Clusters 2 (*n* = 3126, 43.1%) and Clusters 4 (*n* = 1062, 19.4%), in which *bmpr2b*, *gata6*, *bmper*, *smc2*, and *smc5* were overrepresented. It is known that Bmpr2b, a bone morphogenetic protein receptor that can mediate the BMP signaling pathway, plays an indispensable role in the developmental process of the liver [66]. However, functional mechanisms underlying most DEGs, biological processes, and signaling pathways found in the study remain to be further investigated.

## 5. Conclusions

Comparative transcriptome analysis has uncovered a significant difference in hepatocytes between 60–72 hpf and 72–96 hpf in the numbers, types, and expression levels of transcripts. Hepatocytes from 60 to 90 hpf proliferate and establish the functions of lipid synthesis and energy metabolism. Hepatocytes between 60 to 72 hpf developed the function of carbohydrate metabolism. Hepatocytes between 72 to 96 hpf are functionally matured due to the establishment of functions in metabolisms of lipid, protein and energy, cellular secretion, and detoxification. These findings provide novel information to further understand the mechanisms controlling the proliferation and maturation of hepatocytes during liver development. 

## Figures and Tables

**Table 1:** Statistics of RNA-seq output data.

Sample Names	Total Reads (M)	Q20 (%)	Q30 (%)	GC (%)	Read Length (bp) (bp (bp))
60h-1	30.5984 M	92.18	86.74	47.10	114
60h-2	33.1771 M	92.12	86.62	46.76	116
60h-3	37.8892 M	91.96	86.47	46.81	115
72h-1	28.4021 M	92.08	86.67	49.80	110
72h-2	26.1058 M	91.28	86.10	48.58	106
72h-3	30.7598 M	91.99	86.55	49.56	111
96h-1	40.4443 M	92.63	87.45	49.58	107
96h-2	30.7934 M	91.55	86.18	44.64	111
96h-3	27.2249 M	89.38	83.54	46.10	108

Notes: M, million pairs; bp, base pair.

**Table 2:** Top 10 hub pathways of genes included in a and b ranked by MCC method.

Rank	Signaling Pathway	Score
1	Ubiquitin mediated proteolysis	4
1	Cell cycle	4
3	Homologous recombination	2
3	ErbB signaling pathway	2
3	mRNA surveillance pathway	2
3	Hedgehog signaling pathway	2
7	RNA degradation	1
7	Base excision repair	1
7	Fanconi anemia pathway	1
7	Nucleocytoplasmic transport	1

**Table 3:** Top 10 hub pathways of genes included in f and g ranked by MCC method.

Rank	Signaling Pathway	Score
1	Nucleotide excision repair	74
2	Mismatch repair	72
2	DNA replication	72
4	Base excision repair	49
5	Homologous recombination	48
6	Cell cycle	26
7	Fanconi anemia pathway	24
8	p53 signaling pathway	5
9	Herpes simplex virus 1 infection	3
10	Pyrimidine metabolism	2

**Table 4:** Top 10 hub pathways of genes included in c, f, and g ranked by MCC method.

Rank	Signaling Pathway	Score
1	Nucleotide excision repair	102
2	Mismatch repair	96
2	DNA replication	96
4	Cell cycle	54
4	Fanconi anemia pathway	54
6	Base excision repair	49
7	Homologous recombination	48
8	p53 signaling pathway	7
9	Lysosome	3
9	Various types of N-glycan biosynthesis	3

## Data Availability

Data in this study are contained within the article and supplementary material. The sequencing data of this study was submitted to NCBI Sequence Read Archive under BioProject accession number PRJNA849172.

## Supplementary Materials

The following supporting information can be downloaded at: https://www.mdpi.com/article/10.3390/biomedicines10092264/s1, Figure S1: Flow cytometric analysis and the sorting of hepatocytes from embryos at different time points; Figure S2: GO enrichment analysis of genes expressed during different time periods of liver development; Figure S3: Dot plots of KEGG enrichment analysis of genes specifically expressed in hepatocytes at different stages; Table S1: Results of differentially expressed genes in different groups by DEseq2; Table S2: VENN analysis of differentially expressed genes; Table S3: GO enrichment analysis of differentially expressed genes; Table S4: KEGG enrichment analysis of differentially expressed genes; Table S5: Jaccard similarity index of signaling pathways; Table S6: The results of cluster for changes in differentially expressed genes at all three time points.
