# Supplementary figures and images for: Comparative Transcriptome Analysis Provides Novel Molecular Events for the Differentiation and Maturation of Hepatocytes during the Liver Development of Zebrafish
Source: Biomedicines. 2022 Sep 13;10(9):2264. doi: 10.3390/biomedicines10092264 (PMC9496063; doi:10.3390/biomedicines10092264)

Figure S1

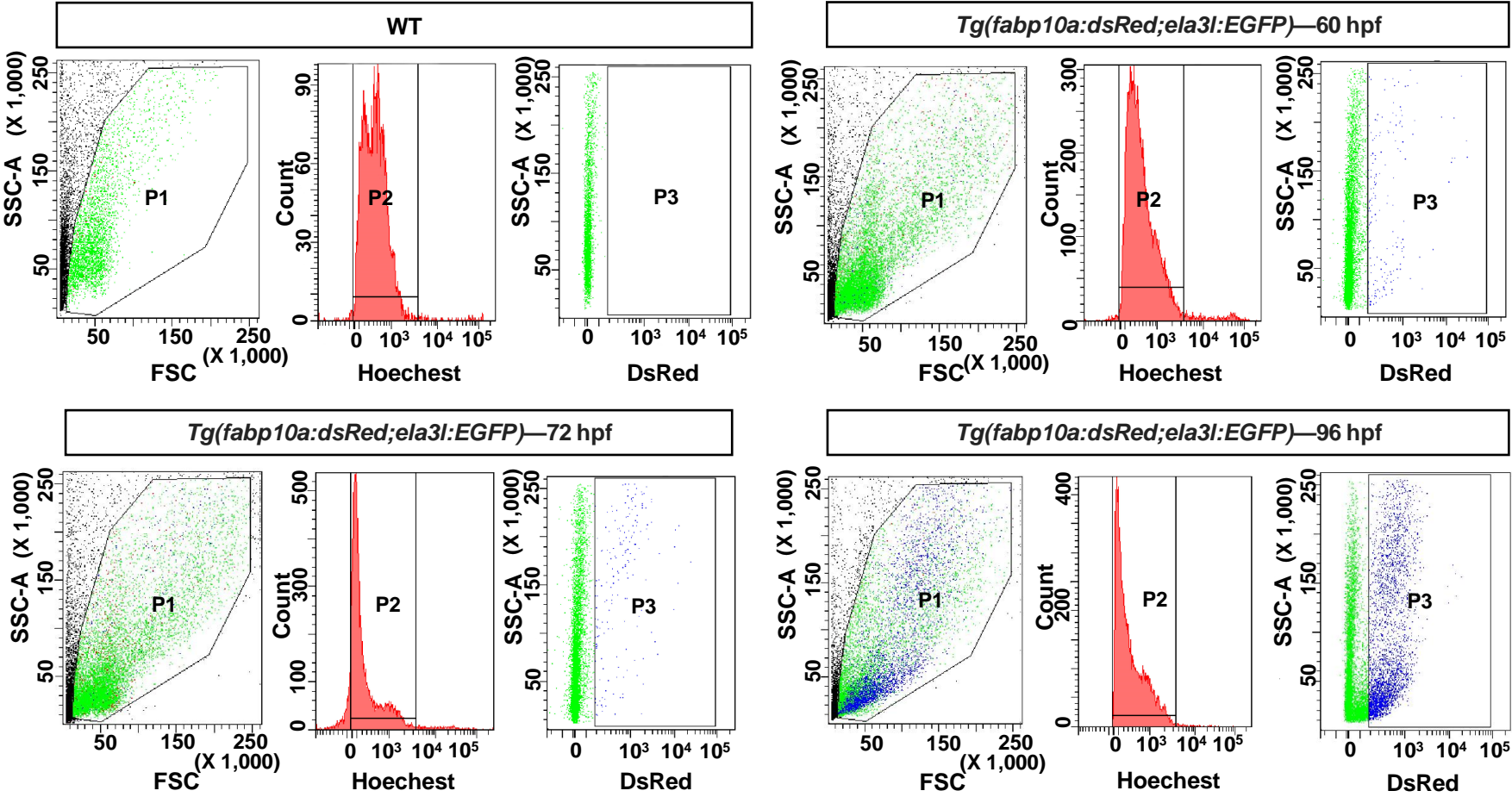

Figure S2

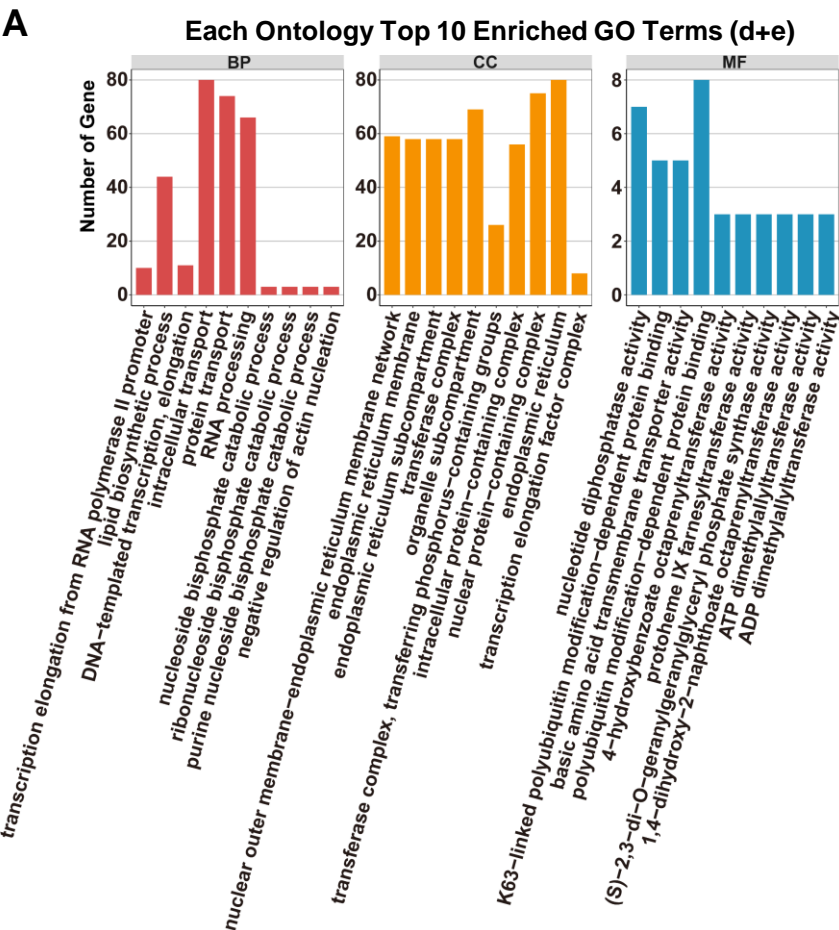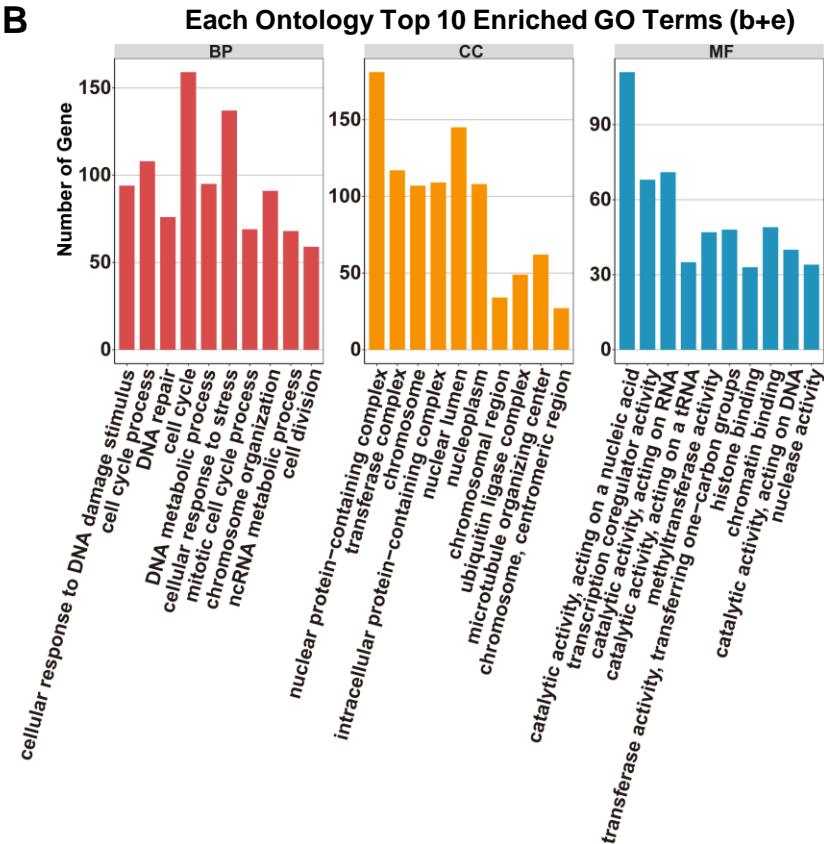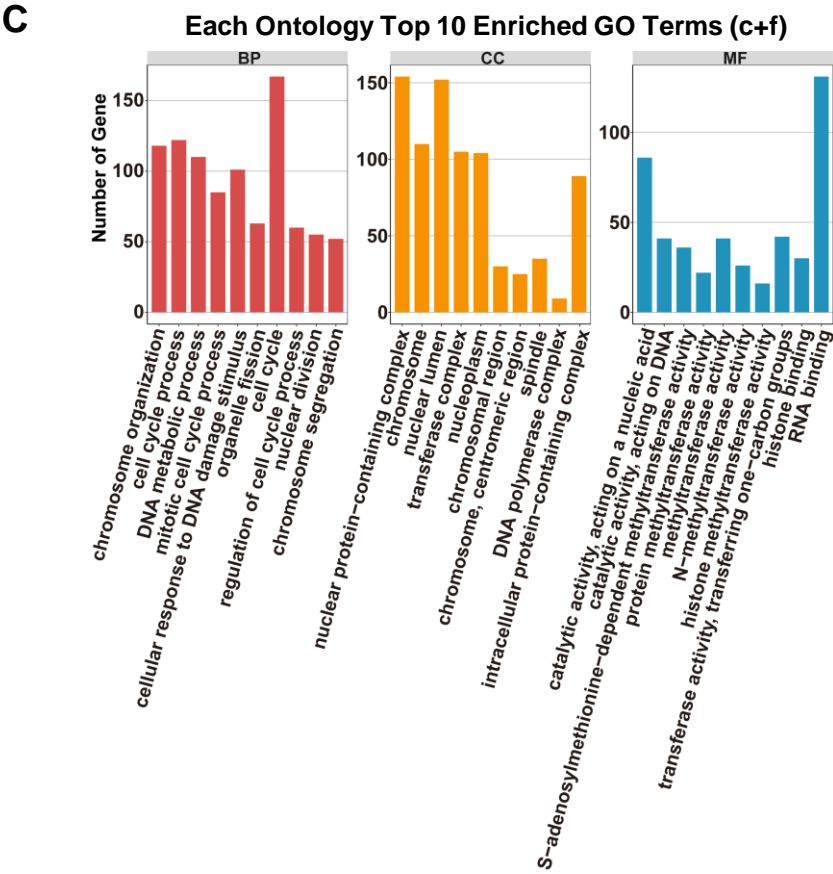

Figure S3

A

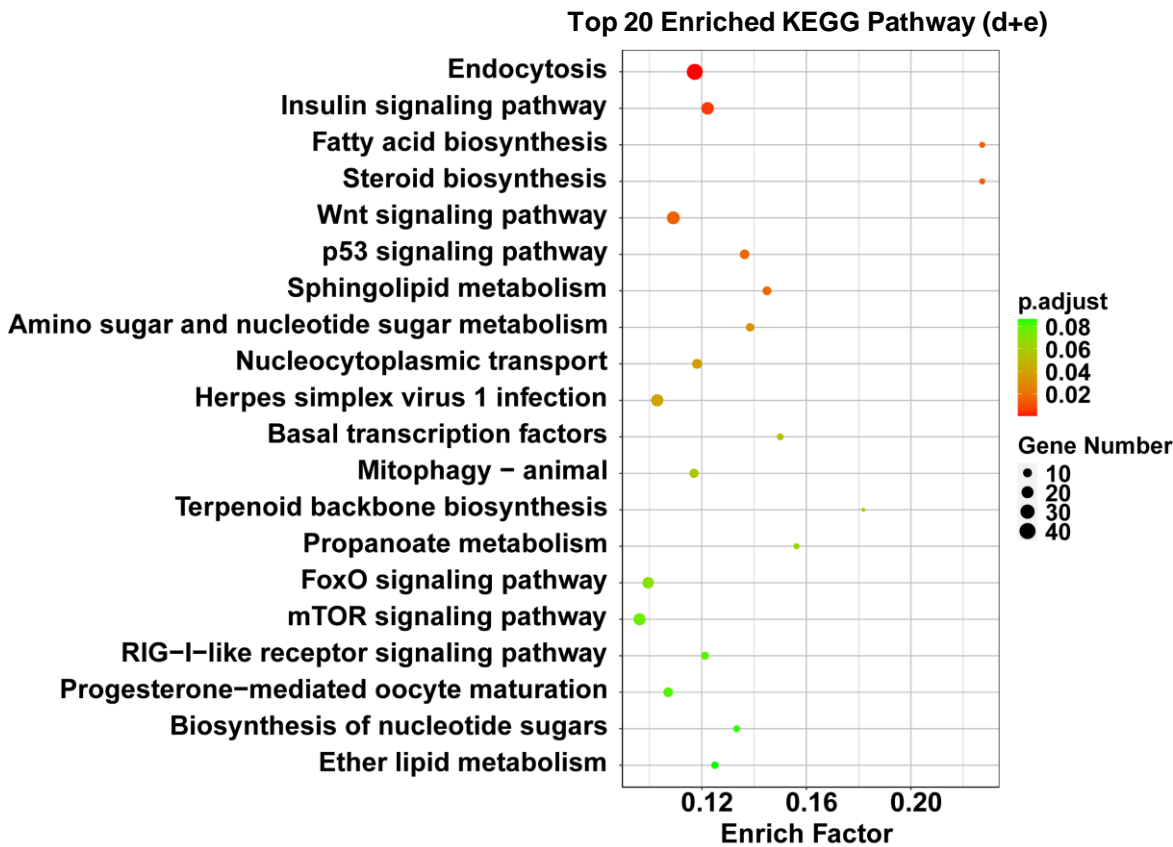

B

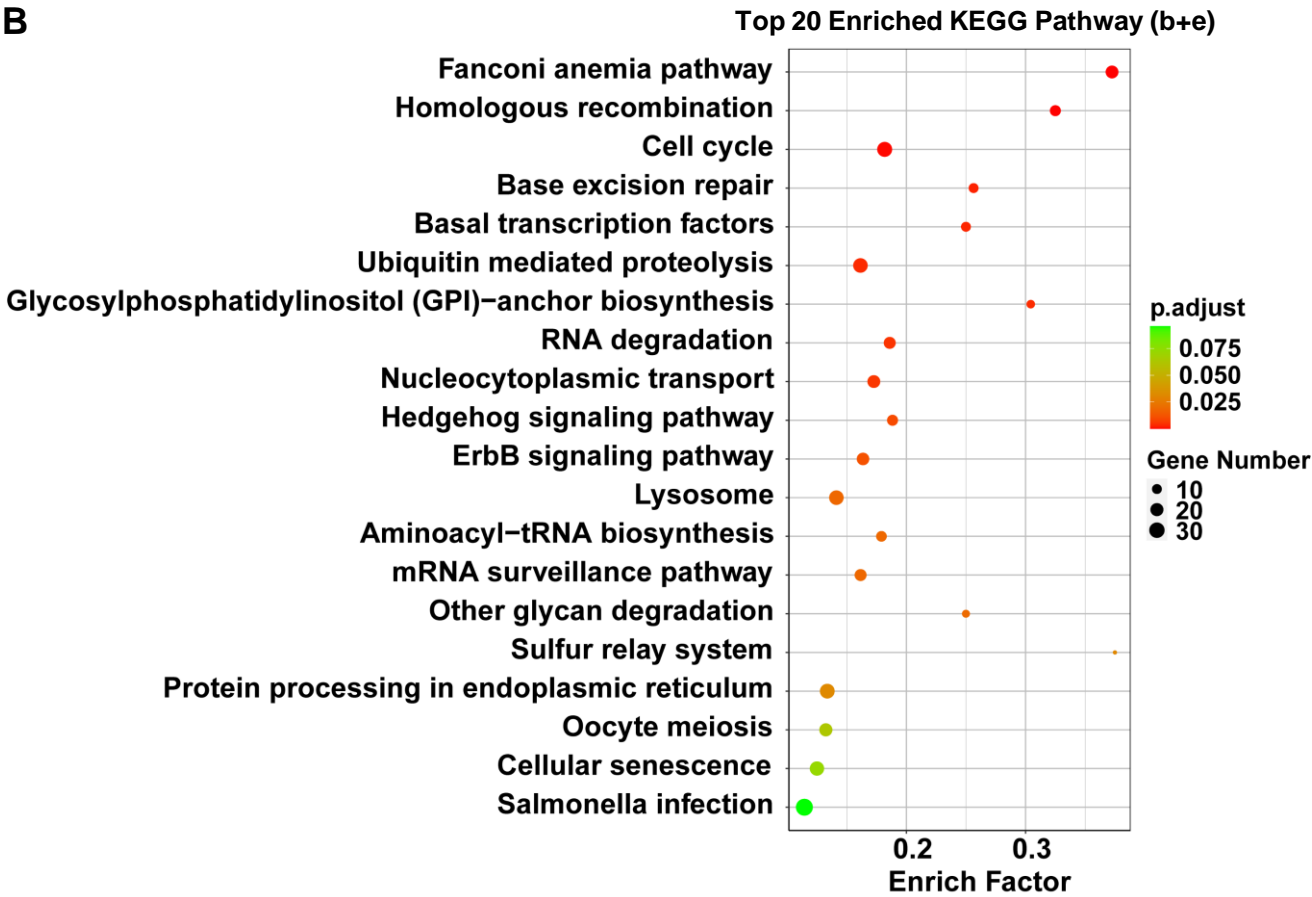

C

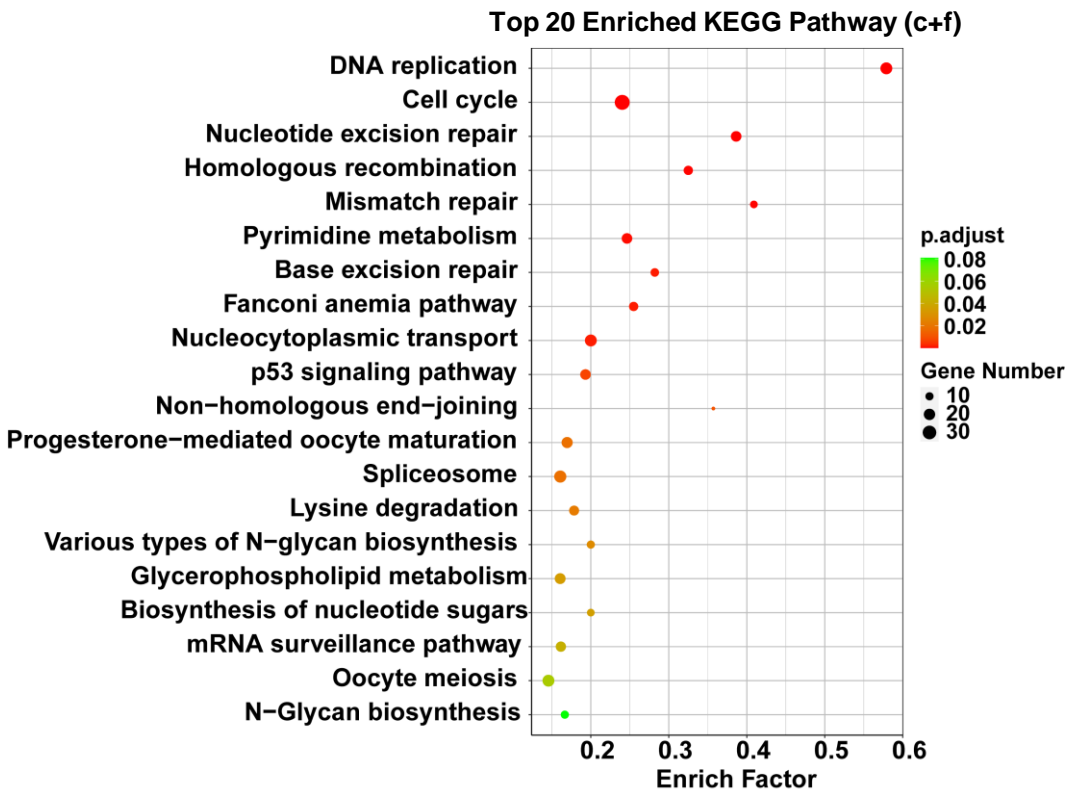

Supplement: Supplementary file 1 [file biomedicines-10-02264-s001.zip › Supplementary Figures-proofreading.pdf]
